# Metabolic regulation of innate immunity in cancer immunotherapy

**DOI:** 10.20892/j.issn.2095-3941.2024.0022

**Published:** 2024-02-05

**Authors:** Yuheng Liao, Hui Yang

**Affiliations:** 1 Department of Neurosurgery, Huashan Hospital, Shanghai Key Laboratory of Medical Epigenetics, International Co-laboratory of Medical Epigenetics and Metabolism (Ministry of Science and Technology), and Molecular and Cell Biology Lab, Institutes of Biomedical Sciences, Institute for Translational Brain Research, Shanghai Medical College of Fudan University, Shanghai 200032, China; 2 Shanghai Key Laboratory of Brain Function Restoration and Neural Regeneration, Shanghai Clinical Medical Center of Neurosurgery, Neurosurgical Institute of Fudan University, Huashan Hospital, Shanghai Medical College, Fudan University, Shanghai 200032, China; 3 State Key Laboratory of Medical Neurobiology and MOE Frontiers Center for Brain Science and MOE Frontiers Center for Brain Science, Institutes of Brain Science, Shanghai Medical College, Fudan University, Shanghai 200032, China

Innate immunity, originally recognized as the primary defense mechanism against pathogenic infections, has also been shown to have an important role in anti-tumor immunity. Host cells recognize cytosolic DNA and RNA, which triggers a cascade of signaling events *via* nucleic-acid sensing receptors, including endosomal Toll-like receptors (TLRs), cytoplasmic cyclic GMP-AMP synthase (cGAS) for double-stranded DNA sensing, and cytoplasmic retinoic acid-inducible gene I (RIG-I) for double-stranded RNA detection. These receptors then recruit adaptor proteins, such as Toll/interleukin-1 receptor/resistance protein (TIR) domain-containing adaptor inducing interferon-β (TRIF), stimulator of interferon genes (STING), and mitochondrial antiviral signaling protein (MAVS), to activate TANK-binding kinase 1 (TBK1). TBK1 phosphorylates interferon regulatory factor 3 (IRF3), leading to its dimerization and nuclear translocation, thereby initiating the expression of type I interferons and inflammatory genes. Activation of these innate immune pathways, especially the cGAS-STING pathway within tumor cells or immune cells, such as dendritic cells (DCs), produce type I interferons and chemokines, which in turn attract more CD8+ T cells against tumors^1^. Emerging evidence suggests that cellular metabolic reprogramming influences or amplifies the production of type I interferons and chemokines mediated by innate immune pathways, ultimately augmenting the anti-tumor effect. This editorial focuses on recent insight into how cellular metabolism regulates the activity of innate immune pathways and the potential for enhancing cancer immunotherapy through metabolic intervention.

## Glucose metabolism regulates innate immune pathways

**Figure 1:**
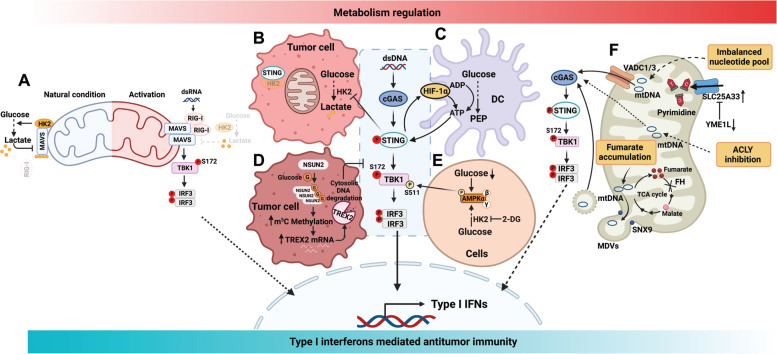
Metabolic regulation of innate immune signaling and mtDNA release. A, Lactate inhibits RIG-I-like receptor (RLR)-mediated interferon production by directly binding MAVS. B, STING inhibits HK2 to restrict tumor aerobic glycolysis and promote antitumor immunity. C, Glycolysis drives STING signaling to augment DC-mediated antitumor immune responses. Mechanistically, HIF-1α–mediated glycolysis is accelerated by DC-intrinsic STING activation and establishes a positive feedback loop. D, Glucose directly binds NSUN2 to suppress cGAS-STING signaling. Mechanistically, the glucose/NSUN2 axis stabilizes TREX2 expression to restrict cytosolic dsDNA accumulation for cGAS-STING activation. E, Glucose deficiency or 2-DG treatment activates AMPK, which directly phosphorylates TBK1(S511) to enhance innate immunity. F, Metabolic control of mtDNA release and the induction of innate immune signaling through fumarate accumulation, nucleotide pool imbalance, or ACLY inhibition. dsDNA/RNA, double strand DNA/RNA; RIG-I, retinoic acid-inducible gene I; MAVS, mitochondrial antiviral signaling protein; HK2, hexokinase II; TBK1, TANK-binding kinase 1; IRF3, interferon regulatory factor 3; STING, stimulator of interferon genes; DC, dendritic cell; HIF-1α, hypoxia-inducible factor-1α; ATP, adenosine 5′-triphosphate; ADP, adenosine diphosphate; PEP, phosphoenolpyruvate; NSUN2, NOP2/Sun RNA methyltransferase 2; TREX2, three prime repair exonuclease 2; m^5^C, RNA 5-methylcytosine; AMPK, AMP-activated protein kinase; 2-DG, 2-deoxy-D-glucose; VDAC, voltage-dependent anion channels; SLC25A33, solute carrier family 25 member 33; YME1L, YME1 Like 1 ATPase; ACLY, adenosine 5′-triphosphate citrate lyase; SNX9, sorting nexin 9; MDVs, mitochondrial-derived vesicles.

Glucose serves as a crucial nutrient for sustaining cellular energy and mass. Glucose is metabolized to pyruvate through glycolysis, then enters the tricarboxylic acid (TCA) cycle *via* the pyruvate dehydrogenase complex (PDHc) in the presence of oxygen or is catalyzed by lactate dehydrogenase (LDH) to generate lactate under hypoxic conditions. Notably, cancer cells exhibit a preference for aerobic glycolysis, a phenomenon known as the ‘Warburg effect,’ resulting in elevated lactate levels. While lactate was traditionally regarded as a byproduct of glycolysis, a paradigm shift occurred in 2019 when lactate was recognized as an intracellular signaling molecule binding directly to the innate immune molecule, MAVS^2^. This interaction disrupts MAVS mitochondrial localization, inhibiting MAVS function, and consequently reducing type I interferon production. Thus, the pharmacologic inhibition of LDHA-dependent lactate production emerges as a promising strategy to enhance the host innate immune response through boosting type I IFN production, thereby strengthening cancer immune surveillance (**Figure 1A**).

Hexokinase II (HK2) serves as the pivotal, rate-limiting enzyme governing the initial step of glycolysis. Recent intriguing findings indicate that cancer cell intrinsic STING inhibits aerobic glycolysis by targeting HK2 to promote anti-tumor immunity^3^. This STING-mediated intervention operates independent of its conventional innate immune role and introduces novel prospects for metabolic therapeutic strategies in specific cGAS-STING-silenced tumor cells (**Figure 1B**). Notably, the influence of glycolysis on the STING pathway extends beyond tumor cells. Hu et al.^4^ reported that tumor-infiltrating DCs exhibit elevated glycolysis, which augments ATP production to enhance the response of the STING pathway, consequently enhancing the production of type I interferon. The augmented activation of the STING pathway, in turn, accelerates hypoxia-inducible factor (HIF)-1α-mediated glycolysis, establishing a positive feedback loop that enhances DC anti-tumor immunity (**Figure 1C**). The intricate interplay between glycolytic metabolism and STING signaling presents a promising opportunity to advance cancer immunotherapies.

In addition to the downstream metabolites, glucose functions as a signaling molecule, regulating the activation of innate immune pathways. A recent study identified NOP2/Sun RNA methyltransferase 2 (NSUN2) as a direct glucose sensor activated by glucose^5^. NSUN2, in turn, maintains the expression of three prime repair exonuclease 2 (TREX2), preventing cGAS-STING activation by degrading cytoplasmic DNA and driving tumorigenesis and immunotherapy resistance (**Figure 1D**). Moreover, diminished intracellular glucose levels activate energy regulator AMP-activated protein kinase (AMPK), which enhances innate immune responses by phosphorylating TBK1^6^ (**Figure 1E**). However, the impact of the AMPK-TBK1 axis on MAVS- and STING-mediated cancer immunology remains unaddressed in this report.

## Metabolites trigger mitochondrial DNA (mtDNA)-dependent innate immune activation

Mitochondria serve as master metabolic hubs, harboring potent inflammatory agonists, such as mtDNA. Mitochondrial stress and damage expose mtDNA to the cytosol, thereby activating the cGAS-STING pathway^7^. The mtDNA-cGAS-STING axis has emerged as a key driver of anti-tumor immunity^8–10^. Metabolic cues induce the release of mtDNA to the cytosol, triggering a type I interferon response (**Figure 1F**). For example, fumarate accumulation triggers the release of mtDNA into the cytosol, activating inflammation through the cGAS-STING pathway^11^. Notably, this process might be mediated by mitochondrial-derived vesicles (MDVs), which involves protein sorting nexin 9 (SNX9) in the budding process. Tumors with fumarate hydratase (FH) mutations, such as hereditary leiomyomatosis and renal cell cancer (HLRCC), exhibit increased immune infiltration. This finding suggests that the inflammatory environment created by mtDNA leakage favors immune infiltration within the tumor microenvironment. Further exploration should focus on whether fumarate levels are correlated with immune infiltration in other tumors. In addition to fumarate, the intracellular nucleotide imbalance mediated by the i-AAA protease, YME1 like 1 ATPase (YME1L) inhibition, also stimulates the release of mtDNA through voltage-dependent anion channels (VDACs) 1/3^12^. Deletion of YME1L leads to the accumulation of the pyrimidine nucleotide carrier, solute carrier family 25 member 33 (SLC25A33), disrupting the balance of pyrimidine transport between mitochondria and cytoplasm, thus driving mtDNA-dependent innate immune responses. This finding raises the intriguing possibility that targeting nucleotide metabolism may limit tumor growth by promoting mtDNA-induced inflammation and the production of type I interferon. Growing evidence highlights the efficacy of activating the cGAS-STING pathway in sensitizing anti–PD-(L)1 therapy-resistant tumors to immunotherapy. Xiang et al.^13^ recently reported that inhibition of adenosine 5′-triphosphate citrate lyase (ACLY) causes polyunsaturated fatty acid (PUFA) peroxidation and mitochondrial damage, triggering mtDNA-mediated cGAS-STING pathway activation. In addition, pharmacologic inhibition of ACLY or dietary PUFA supplementation overcomes cancer resistance to anti–PD-L1 therapy. Overall, understanding the intricate connections between cellular metabolism and mitochondrial programming and how the connections translate into innate immune responses unveils novel avenues for advancing cancer immunotherapy.

## Amino acid metabolic control of innate immunity

Advances in cancer treatment involve dietary modifications, especially amino acids, which offer promise for reprogramming the tumor microenvironment^14,15^. Fang et al.^16^ reported that methionine restriction enhances cGAS-mediated anti-tumor immunity by blocking methylation, which is catalyzed by SUV39H1 histone lysine methyltransferase (SUV39H1). The study highlighted a significant correlation between cGAS methylation and poor prognosis in colorectal cancer patients, suggesting the potential to improve patient outcomes through dietary interventions targeting cGAS methylation. Moreover, limiting serine metabolism increases V-ATPase subunit ATP6V0d2 expression by inhibiting H3K27me3 of its promoter. This inhibition promotes the degradation of yes-associated protein (YAP), lifting the YAP-mediated blockade of TBK1-IRF3 axis, and promoting the production of type I interferon^17^. Although the study involved detailed experiments on antiviral immunity, further research is warranted to determine the impact of serine metabolism on cancer immunotherapy.

Amino acids also have important roles in innate immune cells. Through nutritional screening and comprehensive analysis, Guo et al.^18^ reported that glutamine is the main amino acid that promotes the function of conventional DCs (cDC1s). Supplementing glutamine enhances the anti-tumor effect by promoting the assembly of the folliculin (FLCN)–folliculin interacting protein 2 (FNIP2) complex and inhibiting transcription factor EB (TFEB) activity, thereby boosting the antigen presentation capacity of cDC1s. This effect is impaired when the glutamine transporter solute carrier family 38 member 2 (SLC38A2) is deficient in DC cells. The Guo et al.^18^ study reveals that targeting glutamine acquisition in cDC1s could be a potential strategy for cancer treatment.

## Future perspectives: metabolic interventions in innate immunity have promise for cancer immunotherapy

Recognizing the pivotal role of innate immune pathways in enhancing cancer immunity, numerous endeavors have been undertaken to augment this effect. Metabolic intervention amplifies the response of innate immune pathways, producing more type I interferons and chemokines, thereby recruiting more cytotoxic CD8+ T cells to kill tumors. This approach introduces a new perspective to augment the efficacy of cancer immunotherapy. To further advance this approach, urgent attention is required to identify metabolites or metabolic pathways capable of finely regulating the release of mtDNA. This modulation can sensitize the response of innate immune pathways and be synergistically combined with radiotherapy and chemotherapy for optimal anti-tumor effects. Dietary restriction, as a second avenue, can affect the activity of innate immune molecules (e.g., cGAS) and innate immune cells (e.g., antigen presentation ability). Customizing special diets for patients emerges as a viable strategy to enhance therapeutic outcomes. Furthermore, exploration into metabolites or metabolic enzymes that regulate innate immune pathways or innate immune cells activity unveils potential targets for the development of metabolic inhibitors or agonists to enhance the effect of cancer immunotherapy. For example, targeting immune-responsive gene 1 (IRG1) in tumor-associated macrophages can reverse the immunosuppressive tumor microenvironment, leading to improved immunotherapy outcomes^19^. Similarly, inhibiting 7-dehydrocholesterol reductase (DHCR7) promotes IRF3 activation leading to enhanced IFN-I transcription^20^. Investigating whether a DHCR7 inhibitor holds better therapeutic efficacy in treating cancer patients is a worthy pursuit. However, further studies need to address several existing challenges: how to effectively control the inflammatory storm induced by the innate immune pathway through metabolic regulation; finding the ways to minimize potential side effects of metabolic intervention and exploration of more efficient delivery methods of small molecule drugs targeting metabolism to achieve an optimal treatment effect.
